# Recurrence of generalized cutaneous lichen planus after switching from PD-1 to PD-L1 in a patient with pre-existing oral lichen planus: a case report

**DOI:** 10.3389/fmed.2026.1878585

**Published:** 2026-09-07

**Authors:** Yuning Zhang, Yali Li, Tao Guo

**Affiliations:** 1 Graduate School, Tianjin University of Traditional Chinese Medicine, Tianjin, China; 2 Department of Dermatology, Tianjin Academy of Traditional Chinese Medicine Affiliated Hospital, Tianjin, China

**Keywords:** case report, immune checkpoint inhibitors, immune-related adverse events, lichen planus, PD-1

## Abstract

We present a clinicopathologically documented case of generalized cutaneous lichen planus that recurred after switching from a programmed death 1 (PD-1) inhibitor (camrelizumab) to its ligand PD-L1 inhibitor (atezolizumab) in a patient with pre-existing oral lichen planus. While relapse of induce immune-related adverse events (irAEs) after PD-1/PD-L1 switching has been previously reported, most of these reports described cutaneous manifestations merely as “rash” without detailed dermatological characterization. In contrast, detailed clinical characterization–including clinical photographs, semi-quantitative histopathology, Naranjo causality assessment, treatment response, and long-term follow-up–for a precisely diagnosed cutaneous lichen planus recurrence remains rarely documented. The patient was a 58-years-old female with a 20-years history of oral lichen planus. Following a diagnosis of non-small cell lung cancer (NSCLC), she received camrelizumab. One month later, she developed a generalized cutaneous lichen planus flare, prompting discontinuation of camrelizumab and a switch to atezolizumab. After eight cycles of atezolizumab, the same lichen planus recurred at the identical body sites with greater severity. At admission, the pruritus Visual Analogue Scale (VAS) score was 8. A skin pathology biopsy supported the diagnosis of lichenoid interface dermatitis, with perivascular eosinophil infiltration in the mid-to-deep dermis, consistent with a drug-related etiology. After following treatment with prednisone at a maximum dose of 33 mg, the score decreased to 2. During 10 months of follow-up after discharge, the patient remained stable with no recurrence. This case suggests that recurrence of cutaneous irAEs may occur during sequential use of PD-1 and PD-L1 inhibitors in patients with a history of autoimmune disease.

## 1 Introduction

Immune checkpoint inhibitors (ICIs) have emerged as a novel anti-tumor therapeutic approach, demonstrating significant clinical efficacy in various solid tumors and hematological malignancies. Their core mechanism involves inhibitors of programmed death 1 (PD-1) and programmed death ligand 1 (PD-L1), which block tumor immune evasion and activate the patient’s own immune system, effectively restoring T cell-mediated killing of tumor cells. These agents are widely used not only in non-small cell lung cancer (NSCLC) but also in melanoma, renal cell carcinoma, and many other malignancies. However, it is worth noting that ICI therapy may induce immune-related adverse events (irAEs) due to excessive immune system activation. ICI therapy can trigger flares of various pre-existing autoimmune conditions (e.g., relapsing polychondritis, rheumatoid arthritis, inflammatory bowel disease) and induce *de novo* irAEs across multiple organ systems (1–3). As one of the organs most commonly affected by ICIs, the skin exhibits a diverse range of adverse reactions, including eczema, folliculitis, psoriasis, lichen planus, and reactive cutaneous capillary endothelial proliferation (RCCEP) (4). We found that previous reports have described recurrence of irAEs upon a second ICI treatment (switching between PD-1 and PD-L1 inhibitors), but most only described the skin manifestation as “rash” without providing sufficient details on the specific dermatological phenotype or histopathological findings (5, 6) (Supplementary File 1). Herein, we present a clinicopathologically documented case of generalized cutaneous lichen planus that recurred after switching from a PD-1 inhibitor (camrelizumab) to a PD-L1 inhibitor (atezolizumab) in a patient with pre-existing oral lichen planus. This case suggests that recurrence of cutaneous irAEs may occur during sequential use of PD-1 and PD-L1 inhibitors in patients with a history of autoimmune disease, as switching to an alternative checkpoint target within the same pathway does not necessarily circumvent the risk.

## 2 Case report

The patient was a 58-years-old female with a 20-years history of oral lichen planus, which had been stable for a long time, and she had no prior systemic corticosteroid therapy. She also has a 1-year history of hypertension, for which treated with candesartan, and an 8-months history of elevated blood glucose, treated with acarbose. She was diagnosed with stage IV non-small cell lung cancer in November 2023 and had been treated with camrelizumab as a single agent with no combination chemotherapy. One month after initiating treatment with camrelizumab, according to the patient’s outside medical records and available documentation, the treatment response was stable disease. But she developed erythematous and violaceous flat papules on the trunk and extremities, symmetrically distributed, predominantly on the lower back and lower limbs, accompanied by pruritus. At day 75 of camrelizumab, the erythematous and violaceous flat papules worsened significantly, constituting the first cutaneous flare, and the drug was discontinued immediately thereafter. The erythematous and violaceous flat papules then gradually improved over the next 4 weeks with prednisone 15 mg/day, after which atezolizumab was initiated administered once monthly. After eight cycles of atezolizumab monotherapy, according to the patient’s outside medical records and available documentation, the best response was a partial response. However, the aforementioned erythematous and violaceous flat papules reappeared in the same areas, was more severe than before, and was accompanied by intense itching. Atezolizumab was permanently discontinued after the second flare; no further ICI was administered thereafter and regular follow-up visits with the oncology department are required.

Upon presentation to our dermatology department, physical examination revealed widespread dark erythema on the trunk and extremities, with overlying white reticulated striae (Wickham striae), most severe on both lower limbs and feet (Figure 1A). At the time of the second flare, the oral mucosa showed no active erosions or ulcers (only residual mild hyperkeratosis), and the vulvar mucosa was not involved. The Visual Analogue Scale (VAS) for pruritus was 8, the Naranjo scale score was 7, with the following item scores: (1) Previous conclusive reports on this reaction? +1; (2) Did the adverse event appear after the suspect drug was administered? 0; (3) Did the adverse reaction improve when the drug was discontinued? 0; (4) Did the adverse reaction appear when the drug was re-administered? +2; (5) Alternative causes? 0; (6) Placebo response? +1; (7) Drug detected in blood? 0; (8) Reaction more severe with increased dose? +1; (9) Previous exposure to same drug with similar reaction? +1; (10) Objective evidence? +1. This yielded a total score of 7, indicating a “probable” adverse drug reaction. The Naranjo scale score of 7 was calculated specifically for atezolizumab (the second flare). For the first flare with camrelizumab, formal scoring was not performed before our evaluation; however, the temporal association was also supportive. Histopathological examination of a skin biopsy performed at our hospital revealed parakeratosis, acanthosis, liquefactive degeneration of the basal cell layer (grade 3, affecting approximately 70% of the basal layer), subepidermal cleft formation, and moderate to dense perivascular multifocal infiltration of lymphocytes (estimated density: 80–100 cells per high-power field) extending to the mid-to-deep dermis. Scattered pigment-laden macrophages and eosinophils (approximately 3–4 per high-power field) were observed in the mid-to-deep dermis. Civatte bodies (colloid bodies) were identified at the dermoepidermal junction (Figure 2). Laboratory tests showed no significant abnormalities. Serological tests for hepatitis B (HBsAg, anti-HBs, HBeAg, anti-HBe, anti-HBc) and hepatitis C (anti-HCV) were negative. After consulting with an oncologist, based on the temporal relationship with ICI administration (re-challenge positivity), the characteristic histopathology, the supportive Naranjo score of 7, and the exclusion of other causes, a final diagnosis of drug-induced lichen planus, triggered by immune checkpoint inhibitors, was made. Given that this second episode was markedly more severe than the first, the patient was initially treated with a higher dose of prednisone at 33 mg/day (calculated based on body weight of 66 kg, approximately 0.5 mg/kg/day). By day 8 of high-dose prednisone (33 mg/day), the VAS score decreased from 8 to 2, and the lesions showed visible flattening. The violaceous flat papules gradually faded, leaving dark brown macules (Figure 1B), with occasional pruritus (VAS score 2) (Figure 3). Complete flattening of all papules and resolution of pruritus were achieved after 1 month of prednisone therapy; however, post-inflammatory hyperpigmentation (dark brown macules) persisted at the previous lesion sites (Figure 1B). The prednisone dose was tapered gradually over 8 weeks: 33 mg/day for 5 days, then 30 mg/day for 14 days, 25 mg/day for 14 days, 20 mg/day for 14 days, 15 mg/day for 14 days, 10 mg/day for 14 days and then tapered by 2.5 mg every 5 days until discontinuation. The total duration of corticosteroid therapy was approximately 14 weeks. During 10 months of follow-up, the patient’s oncological disease remained stable, and no further cutaneous flares occurred.

**FIGURE 1:**
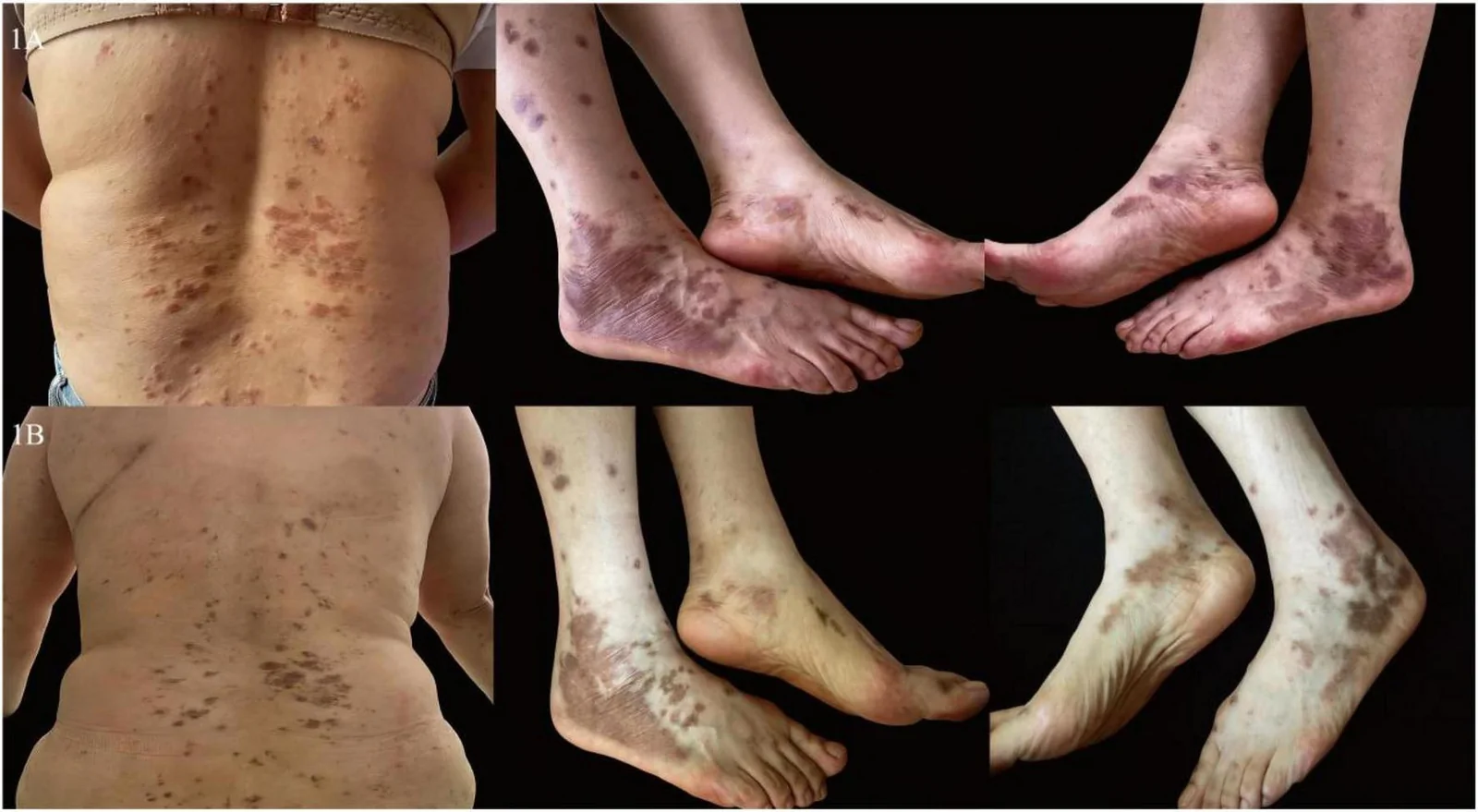
**(A)** Pre-treatment skin lesions: widespread violaceous to dark erythematous flat papules on the trunk and extremities, with overlying white reticulated striae (Wickham striae). **(B)** Post-treatment skin lesions: lesions flattened, with extensive dark brown hyperpigmentation.

**FIGURE 2:**
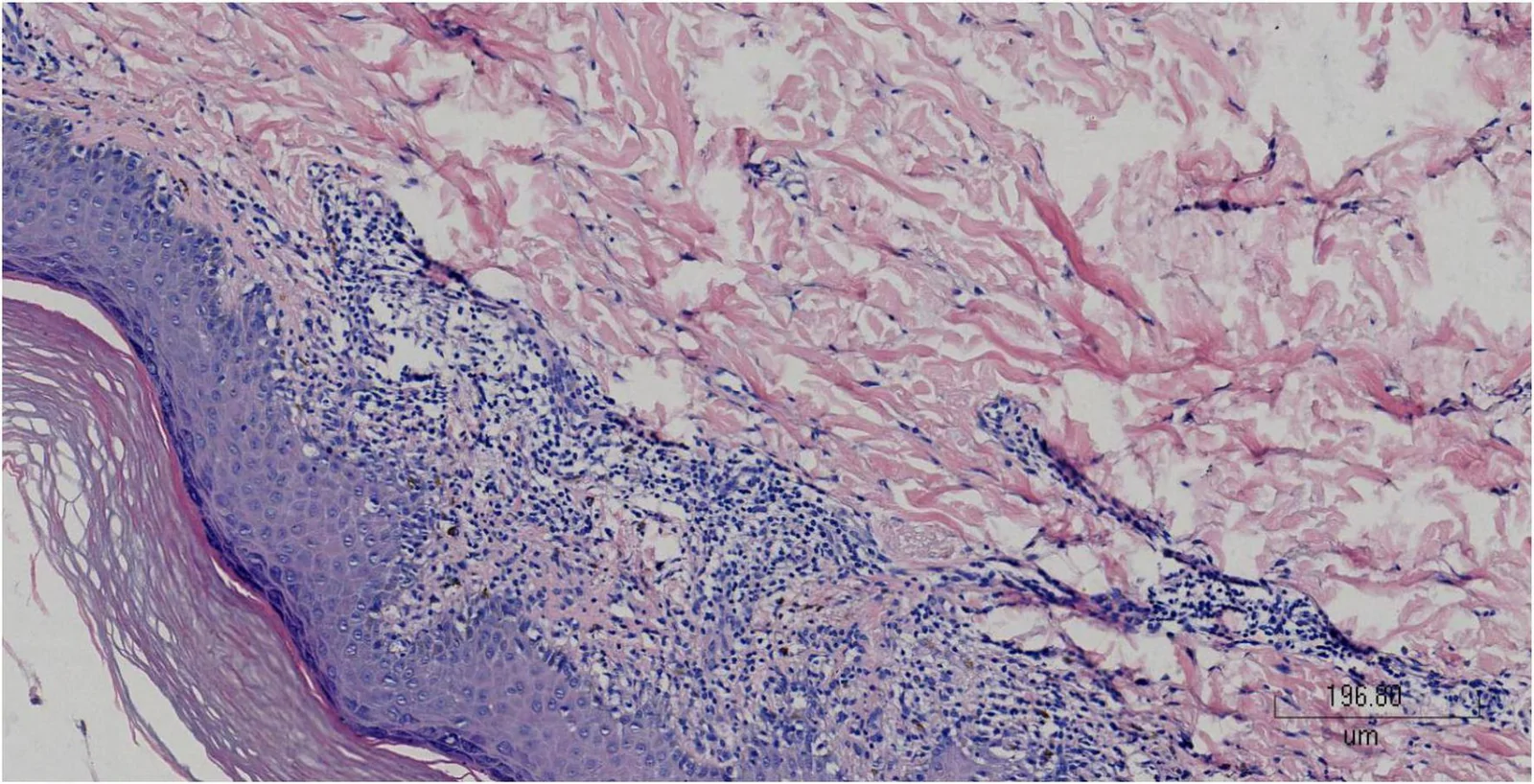
Histopathology: parakeratosis, acanthosis, liquefactive degeneration of the basal cell layer (grade 3, affecting approximately 70% of the basal layer), subepidermal cleft formation, and moderate to dense perivascular infiltration of multifocal lymphocytes (estimated density: 80–100 cells per high-power field) extending to the mid-to-deep dermis. Scattered pigment-laden macrophages and eosinophils (approximately 3–4 per high-power field) were observed in the mid-to-deep dermis. Civatte bodies (colloid bodies) were identified at the dermoepidermal junction. (HE stain, 100×).

**FIGURE 3:**
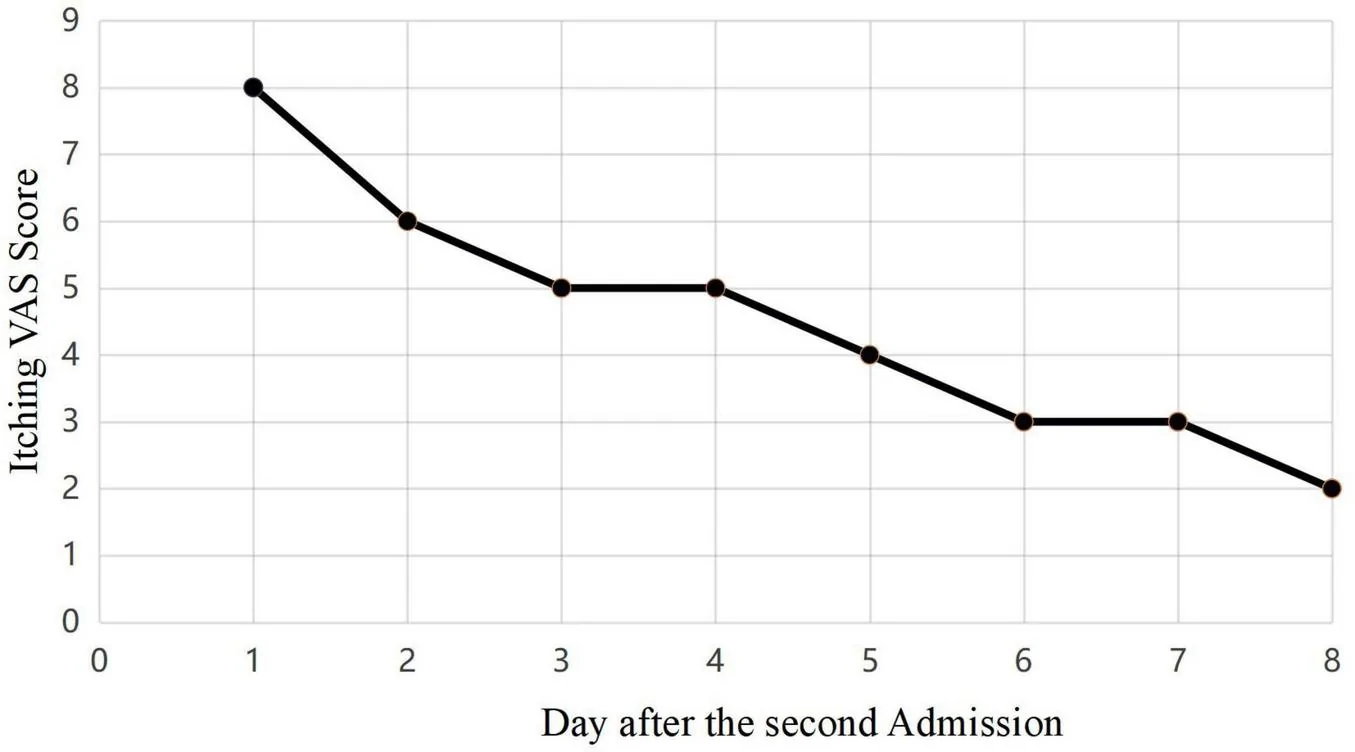
Line chart of itching VAS score changes after the second admission.

## 3 Discussion

In recent years, a number of cases of cutaneous irAEs induced by atezolizumab or camrelizumab have been reported (detailed in Table 1). Among these, atezolizumab has been reported to induce generalized cutaneous lichen planus (7) and lichen planus pemphigoides (8) during treatment for metastatic small cell lung cancer, while camrelizumab has been associated with subacute cutaneous lupus erythematosus and pruritus. Except for two cases of atezolizumab-induced psoriasiform eruptions where patients had a prior history (9, 10), most of these reported cutaneous irAEs occurred in patients with no prior dermatological history. Moreover, previous reports have described recurrence of irAEs upon a second ICI treatment (such as switching between PD-1 and PD-L1 inhibitors). Kitagawa et al. previously reported that among 10 patients with NSCLC who developed irAEs during their first ICI treatment, 4 experienced recurrence of irAEs during a second ICI course after switching between PD-1 and PD-L1 inhibitors, indicating that such switching does not eliminate the risk of irAEs recurrence (6). However, previous reports on cutaneous irAEs after PD-1/PD-L1 switching often described skin manifestations merely as “rash” without sufficient dermatological detail, whereas the present case provides a precisely defined disease entity–generalized cutaneous lichen planus–supported by comprehensive clinical, histopathological, and temporal association evidence.

**TABLE 1:** Review of cases of atezolizumab/camrelizumab-induced cutaneous adverse reactions reported in the literature (7–10, 17–20)*.

Number	References	Drugs	Cutanous diverse reactions	Time between drug introduction and cutaneous diverse reactions	Past relevant medical history
1	Asim et al. (7)	Atezolizumab	Lichen planus pemphigoides	9 months	No
2	Naum et al. (8)	Atezolizumab	Generalized lichen planus	2 months	No
3	Gleason et al. (9)	Atezolizumab	Psoriasiform drug eruption	2 months	Yes, pre-existing psoriasis
4	Fukui et al. (10)	Atezolizumab	Psoriasis vulgaris	6 months	Yes, pre-existing psoriasis
5	Yamada et al. (17)	Atezolizumab	Palmar-plantar erythrodysesthesia	8 months	No
6	Quach et al. (18)	Atezolizumab	Urticarial vasculitis	3 weeks	No
7	Teng et al. (19)	Camrelizumab	Lupus erythematosus	3 weeks	No
8	Watanabe et al. (20)	Camrelizumab	Desquamation, hyperpigmented lesions, and skin breakdown	10 days	No

*This table summarizes cutaneous irAEs reported with atezolizumab or camrelizumab as monotherapy (not necessarily sequential).

While our case involved camrelizumab and atezolizumab, which are commonly used in China, the more widely prescribed PD-1 inhibitors nivolumab and pembrolizumab have also been consistently associated with lichen planus and lichenoid reactions. Importantly, for patients with pre-existing autoimmune conditions, the use of these ICIs carries a well-documented risk of disease flare. A large retrospective study found that 29.5% of cancer patients with pre-existing autoimmune diseases experienced a flare of their original condition after starting ICI therapy (11). The risk of flare varies considerably depending on the underlying autoimmune disease. A systematic review and meta-analysis of over 700 patients with rheumatologic diseases reported an overall flare incidence of 33.8% (95% CI, 25.8–42.2). Among specific conditions, rheumatoid arthritis had a flare incidence of 40.9%, psoriatic arthritis was even higher at 46.4%, while systemic lupus erythematosus had a relatively lower rate of 3.8% (12, 13). Fortunately, the majority of these flares are manageable with systemic corticosteroids, with 81% of treated cases showing clinical improvement; permanent discontinuation of ICIs due to flare occurs in approximately 17% of cases. These findings underscore that lichenoid eruptions and other irAEs represent a class effect of PD-1/PD-L1 blockade, and clinicians should exercise particular caution when treating patients with pre-existing autoimmune diseases.

The clinical course of this case is consistent with the previously described mechanistic framework: the sequential use of PD-1/PD-L1 inhibitors not only restores T cell-mediated tumor killing but may also disrupt T cell immune homeostasis against self-antigens in the skin, leading T cells to attack keratinocytes and other self-antigens, thereby inducing cutaneous adverse reactions (14). Specifically, PD-1/PD-L1 blockade can enhance CD8+ T cell-mediated keratinocyte cytotoxicity through the release of perforin and granzyme B (15). Keratinocytes themselves express PD-L1 under inflammatory conditions, and its blockade may remove an important local inhibitory signal, further facilitating T cell activation (16). Furthermore, patients with pre-existing autoimmune disease may harbor autoreactive T cell clones that are kept in check by PD-1-mediated peripheral tolerance; ICI therapy can release these clones, leading to lichenoid tissue reactions at sites of previous disease activity (21). The recurrence at identical anatomical sites after switching to a different checkpoint inhibitor suggests a local memory T-cell response. It is plausible that tissue-resident memory T cells (TRM), previously activated by the first ICI, remained in the skin and were reactivated upon subsequent PD-L1 blockade, leading to a similar topographic distribution (22). The stepwise escalation of autoimmune activity under sustained ICI pressure supports this mechanism. In this case, each recurrence was more severe than the previous episode, requiring a higher dose of prednisone–from 15 mg/day for the first flare, to 33 mg/day for the second–reflecting a stepwise escalation consistent with increasing autoimmune activity under sustained ICI pressure. Importantly, both flares were sensitive to corticosteroid therapy, suggesting that effective and timely corticosteroid intervention remains a feasible strategy to manage cutaneous adverse reactions while preserving anti-tumor efficacy. Clinicians should appropriately weigh the dual role of corticosteroids in this context.

Our histopathological findings (parakeratosis, deeper dermal infiltration, eosinophils) are more consistent with a drug-induced lichenoid pattern, but the overall clinical presentation supports generalized lichen planus flare triggered by ICI. Notably, Meier-Schiesser et al. demonstrated that ICI-induced lichen planus has distinct clinical, histological, and gene-expression profiles compared with spontaneous lichen planus, supporting the concept of a drug-triggered flare in predisposed individuals (23). Regarding the exclusion of other triggers, neither corticosteroid withdrawal nor candesartan is supported as a cause. The patient did not receive prior systemic corticosteroids for oral lichen planus before initiating ICI therapy; both episodes of rash recurrence were temporally associated with ICI rechallenge rather than with steroid tapering. Candesartan was initiated after the first rash appeared and was continued without interruption thereafter; if it had been causative, the rash would have persisted or recurred earlier. In summary, these factors can be largely ruled out.

Lichen planus and lichenoid drug reactions account for approximately 5%-10% of cutaneous irAEs, though the exact frequency varies with the ICI agent and tumor type. According to the CTCAE v5.0, cutaneous lichen planus is graded as Grade 1 (localized, asymptomatic), Grade 2 (widespread, symptomatic, limiting instrumental ADL), or Grade 3 (severe, limiting self-care ADL). Our patient’s extensive lesions with severe pruritus (VAS 8) correspond to Grade 2. Red flags for severe cutaneous irAEs include rapid progression, mucosal involvement, blistering, or systemic symptoms, which warrant prompt corticosteroid therapy. Differential diagnoses include lichenoid drug eruption, psoriasis, and eczema; histopathology and temporal association are key to distinguish them. It should be emphasized that the above mechanistic discussion is based on theoretical frameworks derived from previous basic and preclinical studies. The temporal association, positive rechallenge, and histopathological features that we present merely provide observational concordance with these known mechanisms at the clinical level, rather than causal verification. Definitive mechanistic attribution awaits larger translational studies in the future.

However, as a case report, this study has limitations. It should be noted that the first cutaneous flare was diagnosed clinically without a skin biopsy. Despite the absence of direct immunofluorescence and specific eosinophil count, which are specific investigations that could affect the diagnostic reliability of lichenoid drug eruption associated with immune checkpoint inhibitors, we believe the diagnosis is well-grounded, as it is supported by qualitative evaluation of the pathological sections, the timing of symptom development, and the overall clinical presentation. As a retrospective case report, the oncological data (TNM stage, RECIST responses) and some medication timing details rely on outside medical records and patient recollection, which may introduce imprecision and affect the certainty of causal attribution. Nonetheless, the core clinical observations–positive rechallenge, characteristic histopathology, supportive Naranjo score, and long-term follow-up–provide valuable evidence for the recurrence risk of cutaneous irAEs upon sequential use of PD-1 and PD-L1 inhibitors in patients with pre-existing autoimmune conditions. Future large-scale cohort studies are needed to further explore how to achieve effective management of cutaneous immune-related adverse events through accurate diagnosis, early intervention, and timely treatment without completely suppressing anti-tumor immunity, thereby achieving individualized treatment goals of “enhanced efficacy and reduced toxicity.”

## 4 Conclusion

We present a clinicopathologically documented case of generalized cutaneous lichen planus that recurred after switching from a PD-1 inhibitor (camrelizumab) to a PD-L1 inhibitor (atezolizumab) in a patient with pre-existing oral lichen planus. This case suggests that recurrence of cutaneous irAEs may occur during sequential use of PD-1 and PD-L1 inhibitors in patients with a history of autoimmune disease. Clinicians should maintain a high index of suspicion for irAE recurrence in this patient population, as switching to an alternative checkpoint target within the same pathway does not necessarily circumvent the risk.

## Data Availability

The original contributions presented in this study are included in this article/Supplementary material, further inquiries can be directed to the corresponding authors.
