# Supplementary material for: Recurrence of generalized cutaneous lichen planus after switching from PD-1 to PD-L1 in a patient with pre-existing oral lichen planus: a case report
Source: Front Med (Lausanne). 2026 Sep 7;13:1878585. doi: 10.3389/fmed.2026.1878585 (PMC13573176; doi:10.3389/fmed.2026.1878585)
Supplement: Supplementary file 1 [file Supplementary_file_1.docx]

To identify published case reports and case series of lichen planus or lichenoid drug eruptions associated with PD-1/PD-L1 inhibitors, with a specific focus on:

Sequential use or switching between PD-1 and PD-L1 inhibitors;Recurrence of cutaneous immune-related adverse events (irAEs) upon rechallenge;Patients with pre-existing autoimmune diseases.

2. Databases Searched

PubMed (MEDLINE)、Web of Science (Core Collection)、Embase (Elsevier)、Cochrane Library (Cochrane Central Register of Controlled Trials)

In addition, we manually cross-checked the following pharmacovigilance databases by consulting publicly available literature and systematic reviews: WHO VigiBase、FDA Adverse Event Reporting System (FAERS)、EudraVigilance (European Union)

3. Search Date

The literature search was performed on February 1, 2026. No lower date limit was applied to ensure comprehensive coverage of all available historical data.

4. Complete Search String (for PubMed, adapted for other databases)

text

("lichen planus" OR "lichenoid drug eruption" OR "lichenoid dermatitis" OR "lichenoid reaction")

AND

("immune checkpoint inhibitors" OR "PD-1" OR "PD-L1" OR "nivolumab" OR "pembrolizumab" OR "camrelizumab" OR "atezolizumab" OR "durvalumab" OR "avelumab" OR "cemiplimab")

AND

("adverse event" OR "toxicity" OR "skin" OR "cutaneous" OR "irAE" OR "immune-related" OR "dermatologic")

5. Additional Focused Search for Sequential Use and Recurrence

To specifically address the switching/rechallenge context of our case, we performed a supplementary search combining the above terms with the following keywords:

text

("sequential" OR "switch" OR "switching" OR "rechallenge" OR "retreatment" OR "recurrence" OR "flare")

6. Inclusion and Exclusion Criteria

Criteria	Description

Inclusion	- Human studies (case reports, case series, observational studies, and systematic reviews).

- Studies reporting lichen planus, lichenoid drug eruption, or lichenoid dermatitis as a cutaneous irAE.

- Studies involving PD-1, PD-L1, or CTLA-4 inhibitors.

- Reports explicitly mentioning sequential therapy or recurrence after switching ICIs.

- Full-text available in English or Chinese.

Exclusion	- Reviews, editorials, conference abstracts without original patient data.

- Non-dermatological irAEs (e.g., pneumonitis, colitis without skin involvement).

- Studies where the skin manifestation was reported only as "rash" without histopathological or clinical phenotype specification.

- Preclinical studies, animal models, and in vitro experiments.

7. Summary of Evidence

The retrieved literature confirmed that:

Lichen planus and lichenoid reactions are well-documented cutaneous irAEs associated with multiple ICIs, including nivolumab, pembrolizumab, camrelizumab, and atezolizumab.

Recurrence of cutaneous irAEs after switching between PD-1 and PD-L1 inhibitors has been observed, albeit with limited detailed dermatological characterization.

Patients with pre-existing autoimmune conditions (e.g., psoriasis, rheumatoid arthritis, inflammatory bowel disease) are at an elevated risk of disease flare upon ICI initiation, with reported flare incidences ranging from 29.5% to 33.8% in large meta-analyses.
